# Postmortem skin microbiome signatures associated with human cadavers within the first 12 h at the morgue

**DOI:** 10.3389/fmicb.2023.1234254

**Published:** 2023-07-26

**Authors:** Lavinia Iancu, Azdayanti Muslim, Shafiq Aazmi, Victor Jitaru

**Affiliations:** ^1^Department of Criminal Justice, University of North Dakota, Grand Forks, ND, United States; ^2^Department of Medical Microbiology and Parasitology, Faculty of Medicine, Universiti Teknologi MARA, Sungai Buloh Campus, Jalan Hospital, Selangor, Malaysia; ^3^Institute for Biodiversity and Sustainable Development, Universiti Teknologi MARA (UiTM), Selangor, Malaysia; ^4^Microbiome Health and Environment (MiHeaRT), Faculty of Applied Sciences, Universiti Teknologi MARA, Selangor, Malaysia; ^5^School of Biology, Faculty of Applied Science, Universiti Teknologi MARA, Selangor, Malaysia; ^6^Institute of Legal Medicine, Iași, Romania

**Keywords:** skin microbiome, decomposition, cause of death, PMI, morgue

## Abstract

**Introduction:**

Forensic microbiome studies expanded during the last decade, aiming to identify putative bacterial biomarkers to be used for the postmortem interval (PMI) estimation. Bacterial diversity and dynamics during decomposition are influenced by each individual’s micro and macroenvironment, ante and postmortem conditions, varying across body sites and time. The skin, the largest organ of the human body, hosts a diverse microbial diversity, representing the first line of defense of a living individual. Targeting the investigation of the postmortem skin microbiome could help understanding the role of microbes during decomposition, and association with the ante and postmortem conditions.

**Methods:**

The current study aimed to identify the postmortem skin microbiome signatures associated with eight human bodies, received at the Institute of Legal Medicine Iasi, Romania, during April and May 2021. A total of 162 samples (including triplicate) representing face and hands skin microbiome were investigated via Illumina MiSeq, upon arrival at the morgue (T0) and after 12 hours (T1).

**Results:**

The taxonomic characteristics of the skin microbiota varied across different body sites. However, there were no significant differences in taxonomic profiles between collection time, T0 and T1, except for some dynamic changes in the abundance of dominant bacteria. Moreover, different microbial signatures have been associated with a specific cause of death, such as cardiovascular disease, while an elevated blood alcohol level could be associated with a decrease in bacterial richness and diversity.

**Discussion:**

The places where the bodies were discovered seemed to play an important role in explaining the bacterial diversity composition. This study shows promising results towards finding common postmortem bacterial signatures associated with human cadavers within the first 12h at the morgue.

## Introduction

In the current context of forensic science, the term forensic microbiology is typically defined as the study of microorganisms, including their DNA and other biomolecules, to aid in the investigation of crimes or other legal cases. However, in the realm of bioterrorism, forensic microbiology encompasses the illegal deployment of biological agents capable of releasing toxins with dangerous effects on the population (Budowle et al., 2003).

An emerging branch of forensic microbiology, developed in recent years, involves the investigation of microbes associated with decomposition (Iancu et al., 2015, 2018; Javan et al., 2016; Metcalf et al., 2017; Singh et al., 2018), since it is well known that microbes are the driving factor of decomposition and pathogenic spread (Damann et al., 2015; Hauther et al., 2015; Johnson et al., 2016; Adserias-Garriga et al., 2017; DeBruyn and Hauther, 2017; Harrison et al., 2020). In this regard, studies used pig carcasses (Iancu et al., 2015; DeBruyn and Hauther, 2017; Forger et al., 2019), rat carcasses (Metcalf et al., 2013; Guo et al., 2016), and human cadavers (Humphreys et al., 2013; Hauther et al., 2015; Finley et al., 2016; Jeong et al., 2016) exposed in various geographical locations and seasons, to investigate the microbiome patterns during decomposition. The intended application of the forensic microbiome investigation is represented by the postmortem interval (PMI) estimation (Metcalf et al., 2013; Belk et al., 2018). Most of the studies related to the postmortem microbial diversity from human bodies were performed in the USA, primarily in Michigan (Pechal et al., 2018), Texas (Hyde et al., 2013), Alabama (Can et al., 2014), and Tennessee (Damann et al., 2015; Hauther et al., 2015; Johnson et al., 2016; Adserias-Garriga et al., 2017; DeBruyn and Hauther, 2017), while scarce studies were performed in Europe. To emphasize bacteria as “postmortem clocks,” several studies (Hyde et al., 2013; Bucheli and Lynne, 2016) addressed the species/community’s identification and their succession. As previously demonstrated, the postmortem microbiome presents predictable patterns associated with different decomposition stages (Bucheli and Lynne, 2016); however, the specificity of the microbiome related to each individual and antemortem conditions must be taken into consideration. In addition to the individual microbiome characteristics, the diversity and dynamics varies among body locations (Javan et al., 2019). Thus, in our study we chose to sample and investigate face and hand areas, as these are the most exposed to exogenous factors; intending to distinguish common trends among all of this diversity.

A few studies (Pechal et al., 2018; Kodama et al., 2019; Ahannach et al., 2021; Tozzo et al., 2022) aimed to investigate the postmortem microbiome of human skin, as a non-invasive collection method, but also in aiming to associate skin bacteria with personal objects (Gouello et al., 2021; Speruda et al., 2022). These studies revealed that the decomposition microbiome may be linked to antemortem conditions (Pechal et al., 2018). Although these previous studies stated that bacterial decomposition indicators may not be present in all cases and ubiquitous species are not particularly useful for specifying the manner and/or cause of death, the current study aims to propose the usage of ubiquitous microbes and their relative abundances as markers of the PMI, precisely given their cosmopolite presence that can be quantified and related to antemortem and/or postmortem conditions. Considering that each person hosts a different microbiota, composed of up to 1,000 different bacteria species, which in turn varies across body sites and time (Turnbaugh et al., 2007), and that the discriminatory power of the microbiome could be similar to that of a fingerprint (Costello et al., 2009; Grice et al., 2009; Fitz-Gibbon et al., 2013), investigating common taxa/colonization patterns could lead to a better understanding of these variations after death.

Since skin is the most extensive human organ, harboring unique niches of microbes varying from site to site (Costello et al., 2009), and taking into consideration a non-invasive collection technique (via sterile swabs) the current research aimed to investigate skin postmortem microbial signatures. Moreover, since scarce studies aimed to investigate the link between microbiome and cause and manner of death, our studied intended to provide critical information related to the human postmortem skin microbiome signatures along the first 12 h since morgue receiving, by investigating not only the diversity, but also the association with various antemortem conditions. To obtain an overview of the postmortem microbial patterns, skin samples associated with the face and hands of eight human bodies received at the morgue, in Iași (Romania), were investigated during April and May 2021. Due to the highly conserved regions, 16S rRNA is widely used as a genetic marker for the identification of microbial communities (Roy et al., 2021). Moreover, as previously observed (Bell et al., 2018), Next Generation Sequencing (NGS) is best suited for the identification of microbial signatures. Hence, in the current study, the collected skin samples were investigated via NGS Illumina MiSeq.

Decomposition starts from the gastrointestinal tract driven primarily by the lack of response from the immune system and by a low level of oxygen within the cells. While thanatomicrobiome profiles (Javan et al., 2019) are exhibiting correlations with different decomposition stages, skin postmortem microbiomes could reveal similar characteristics for the early postmortem interval. Although necrobiome studies expanded in recent years (Javan et al., 2016, 2019; Oliveira and Amorim, 2018) aiming to help in the PMI estimation, many more studies need to be developed, to better understand the skin postmortem microbiome; given the specificity of each individual’s microbiota. In this context, our research focused on examining the skin postmortem microbiome, with the aim of identifying similarities that would help understand microbial signatures throughout early decomposition.

Our research theory was founded on the following research questions aiming to provide data that could help in elucidating the role of microbes as PMI estimators: (a) are postmortem skin microbiome patterns similar in regard to the human cadavers arrived at the morgue within the first 12 h? (b) if variations are detected, are those triggered by the cause of death or by the antemortem medical conditions? (c) can shared microbial taxa be used as PMI indicators?

## Materials and methods

### Skin microbiome sampling protocol

The skin postmortem microbiome signatures from eight cases representing eight distinct human bodies (referred to from now on as cases F1 and M1–M8) received at the Institute of Legal Medicine Iași (Romania) were investigated during the months of April and May 2021. Samples from case M5 were excluded from the bioinformatics investigation due to the limited technical replicate of the data to represent right hand and face areas. The limited technical replicate was found to affect the univariate and multivariate analysis when using *METAGENassist* (Arndt et al., 2012).

Samples collection was non-invasive and was performed in triplicate via sterile cotton swabs. Three body areas were selected for the investigation: left inner hand (palm), right inner hand (palm), and face (including forehead and cheeks), since these areas are the most exposed to the environment. Swabbing was performed for approximately 30 s with 50 swab rotations /each collection area. The male-to-female ratio was 7:1 and the decedents were of different ages, ranging from 41 to 88 years (Table 1). The location of the body discovery varied from health facility (hospital) to a house in a rural area, and the medical records showed medical conditions such as kidney diseases, dementia, to lower extremity amputations (Table 1). When available, the cause of death, necropsy findings, and toxicological report were considered to better understand the postmortem changes of the skin microbiome patterns (Table 1).

**Table 1:** Metadata table for cases F1 and M1–M8.

Case	Sex	Age	Place of body discovery	Medical records	Cause of death	Necropsy findings	Toxicology report
*F1*	F	86	Health facility (hospital)	Dementia	Myocardial fibrosis	Left ventricular hypertrophy, myocardial fibrosis, coronary atherosclerosis	NA
*M1*	M	65	At home (apartment building)	Ethanol consumer, kidney diseases	Acute necrotizing hemorrhagic pancreatitis	Sternal fracture, cardiomegaly, myocardial fibrosis, coronary atherosclerosis, liver steatosis, hemorrhagic pancreatitis	Blood alcohol level 0 g%
*M2*	M	56	Stairway (apartment building)	Ethanol consumer, heart disease, liver cirrhosis	NA	NA	NA
*M3*	M	57	At home (house)	Amputated calves	Myocardial fibrosis	Pleural adhesions, coronary atherosclerosis, myocardial fibrosis	Blood alcohol level 0.85 g%, alcohol urea 1.12 g%
*M4*	M	70	Health facility (hospital)	Dementia, dilated cardiomyopathy, permanent atrial fibrillation, heart failure, *Clostridium difficile* colitis, deficiency anemia	Myocardial fibrosis	Cerebral atherosclerosis, left ventricular hypertrophy, myocardial fibrosis	NA
*M6*	M	50	At home (apartment building)	NA	Myocardial fibrosis	Bilateral calf edema, scleral jaundice, right pleural liquid effusion, myocardial fibrosis, coronary atherosclerosis, acute pancreatitis, chronic pancreatitis, glomerulosclerosis	Blood alcohol level 0 g%
*M7*	M	41	Health facility (hospital)	Hospitalized for 3 h, resuscitated	Myocardial fibrosis	Putrefaction, myocardial fibrosis, coronary atherosclerosis, hepatic steatosis	Blood alcohol level 0.96 g%
*M8*	M	47	At home (house)	NA	Myocardial fibrosis	Putrefaction, myocardial fibrosis, coronary atherosclerosis, hepatic steatosis	Blood alcohol level 1.81 g%

The sample collection was performed twice over a period of 12 h, with T0 representing the first collection time upon immediate arrival at the morgue, and T1 representing the second collection time, 12 h later. At all times (arrival-first sampling, and second sampling), the cadavers were stored at room temperature; while data on the time elapsed since death and the collection of samples was not available to be included in the metadata table and analyses.

The study received the approval from the research Ethics Committees of the Institute of Legal Medicine Iași (no. 26067/03.23.2021) and the Institutional Biosafety Committee (IBC) University of North Dakota (UND; no. IBC-202111-011).

### Molecular investigation

Immediately after collection, the swab samples were stored at −20°C until further investigation. At the end of the experimental investigation, the samples were shipped on ice from the Institute of Legal Medicine Iași (Romania) to the UND, Forensic Science Laboratory (United States).

Total genomic DNA isolation was performed via Qiagen Blood and Tissue modified protocol, by using the double quantity of the lysis buffers, and stored at −20°C. DNA concentration and purity were assessed via NanoDrop One spectrophotometer (Thermo Scientific, United States). The purity of DNA was determined using the ratio of absorbance at 260 and 280 nm. All experimental steps (isolation, molecular assays) were performed under aseptic conditions to avoid any microbial cross contamination, via a purifier filtered PCR enclosure (Labconco, United States).

The DNA integrity was determined using 1% agarose gel electrophoresis (Sigma-Aldrich, United States) and stained with SYBR Safe DNA Gel Stain (Invitrogen, United States). Extracted DNA was stored at −20°C pending sequencing analysis.

### 16S rRNA gene sequencing

PCR amplification of the 16S rRNA gene fragments (V3–V4 variable region) was performed using the primer pair 357F/806R (University of Minnesota, Genomics Centre, United States). The barcoded amplicons were analyzed in triplicate, pooled in equal concentrations, and sequenced using one lane of the Illumina MiSeq PE300 sequencing platform at the University of Minnesota, Genomics Centre, with a mean read pairs per sample of 91,189. The datasets generated and analyzed during the current study were deposited in the NCBI SRA Sequence Read Archive under the BioProject: PRJNA940997.

### QIIME2—pre-processing and quality filtering

The fastq sequence data was imported into the Quantitative Insights into Microbial Ecology 2 (QIIME2), version 2019.7 (Caporaso et al., 2010; Bolyen et al., 2019). Samples with low sequence read (<1,000) were filtered out using q2-demux. Denoising, phiX chimera removal, and identification of Amplicon Sequence Variants (ASV) tables were performed using DADA2 (Callahan et al., 2016). Primer sequences were trimmed, and the forward and reverse reads were truncated at 260 bases, where the quality scores dropped. The ASV feature data was then rarefied to 49,578 reads per sample (q2-feature table plugin: rarefy; Weiss et al., 2017) and aligned with mafft alignment to construct a phylogeny tree (q2-phylogeny plugin) using FastTree (Price et al., 2010).

### Taxonomic, alpha and beta analyses

The ASV were taxonomically classified using a q2-feature classifier against the pretrained SILVA SSU Ref database (version 132; Quast et al., 2012) with 99% OTUs reference sequences that had been trimmed for the 16S rRNA V3-V4. ASVs identified as chloroplasts or mitochondria were removed from the representative sequences and feature table before any further analyses were conducted. Taxonomic downstream analyses were conducted at phyla, family, and genus levels. Alpha diversity and beta diversity analyses were performed using Shannon’s diversity index (Chao and Shen, 2003) and Bray-Curtis index (Beals, 1984), respectively as implemented in q2-diversity and emperor plugins. A Kruskal-Wallis pair-wise statistic was used for the Shannon index to test the alpha group of significance and the difference between groups. While for Bray-Curtis, all matrices were represented as principal coordinates analysis (PCoA), PERMANOVA tests with 999 permutations were used to determine if the distances between groups were significantly different (Anderson, 2017).

### METAGENassist

Further comparative metagenomics analysis was performed using METAGENassist (Arndt et al., 2012). The skin microbiome was compared between cases, collection time (T0 vs. T1) and cause of death (acute necrotizing hemorrhagic pancreatitis vs. myocardial fibrosis). The inherent differences in depth of sequencing data within metagenomes were normalized using row-wise normalization by the median. Column-wise normalization by autoscaling was employed to obtain a more normal/Gaussian distribution of each relative bacterial abundance.

Univariate statistics such as fold change (FC) analysis (threshold set at 2.0), paired-samples *t*-test, independent samples *t*-test and ANOVA with *Post hoc* Tukey test with a significant *p*-value < 0.05 were used to determine any significant differences in the abundance of each phylum and genera between comparison parameters (Abdullah et al., 2022). Multivariate analysis using the supervised model Partial Least Square Discriminant Analysis (PLSDA) of *β*-diversity was used to reveal any variation in skin microbiome according to the research questions stated in the introduction. The performance of the discriminant pattern from the PLSDA model was evaluated based on R^2^ values (less than 0.33, weak; 0.33–0.67, moderate; 0.67 and above is a substantial model; Peng and Lai, 2012). The significance value of the PLSDA was determined using leave one out cross-validation (LOOCV) with a 1,000 permutation test with a *p*-value < 0.05. The loading plots from PLSDA and variable importance in projection (VIP) were used to determine the importance of each phylum and genus in each community profile (Abdullah et al., 2022). The key genera with VIP scores over 1.0 were considered important contributors to the clustering of microbiota structure observed in the PLSDA model (Akarachantachote et al., 2014).

## Results

Of the eight cases, we successfully generated a total of 7,951,382 high-quality merged sequence reads [median (IQR): 49,578 (33,136-69,377)], which were then mapped to 16,555 amplicon sequence variants (ASVs).

### Alpha and beta diversities

**Figure 1:**
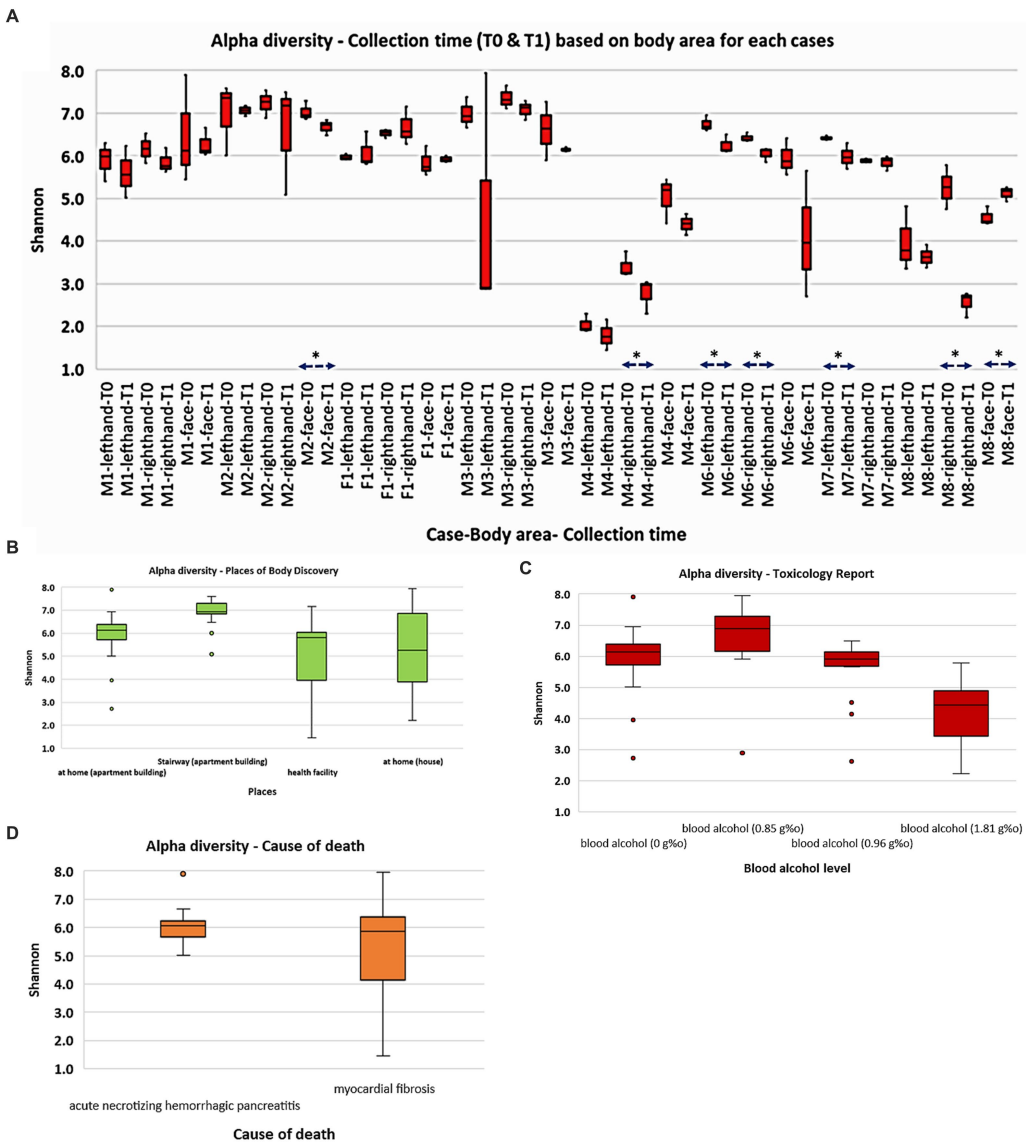
Overall abundance and alpha diversity of skin microbiota based on cadaver cases and selected categories: **(A)** collection time (T0 and T1); **(B)** places of body discovery; **(C)** toxicology report; **(D)** cause of death. Significant different (*p* < 0.05) was marked as *.

Alpha (α) diversity analyses (Figure 1) were utilized with the Shannon index to evaluate the abundance and diversity of the postmortem skin microbiota. The results indicate that the M4 (death recorded at health facility) and M8 (death recorded at home) cases exhibited significantly lower bacterial richness and diversity than the other cases (*p* < 0.001; Kruskal Wallis). Furthermore, M2, whose body was found in the apartment stairway, exhibited significantly higher bacterial communities’ abundance (*p* < 0.05) than most other cases, except for M3. While no significant differences were observed between the face, right hand, and left hand of M1 and M2 cases, we noted dynamic patterns in other cases concerning body sites (*p* < 0.05). Interestingly, we observed a decrease in microbial communities at the time of sampling from T0 to T1 in the majority of the sites, although not all of them exhibited statistically significant reductions. Our observations also suggest that an elevated blood alcohol level may be associated with a decrease in bacterial richness and diversity (*p* < 0.00 L). Other Shannon analyses stratified by the places of body discovery and cause of death are also shown in Figure 1.

**Figure 2:**
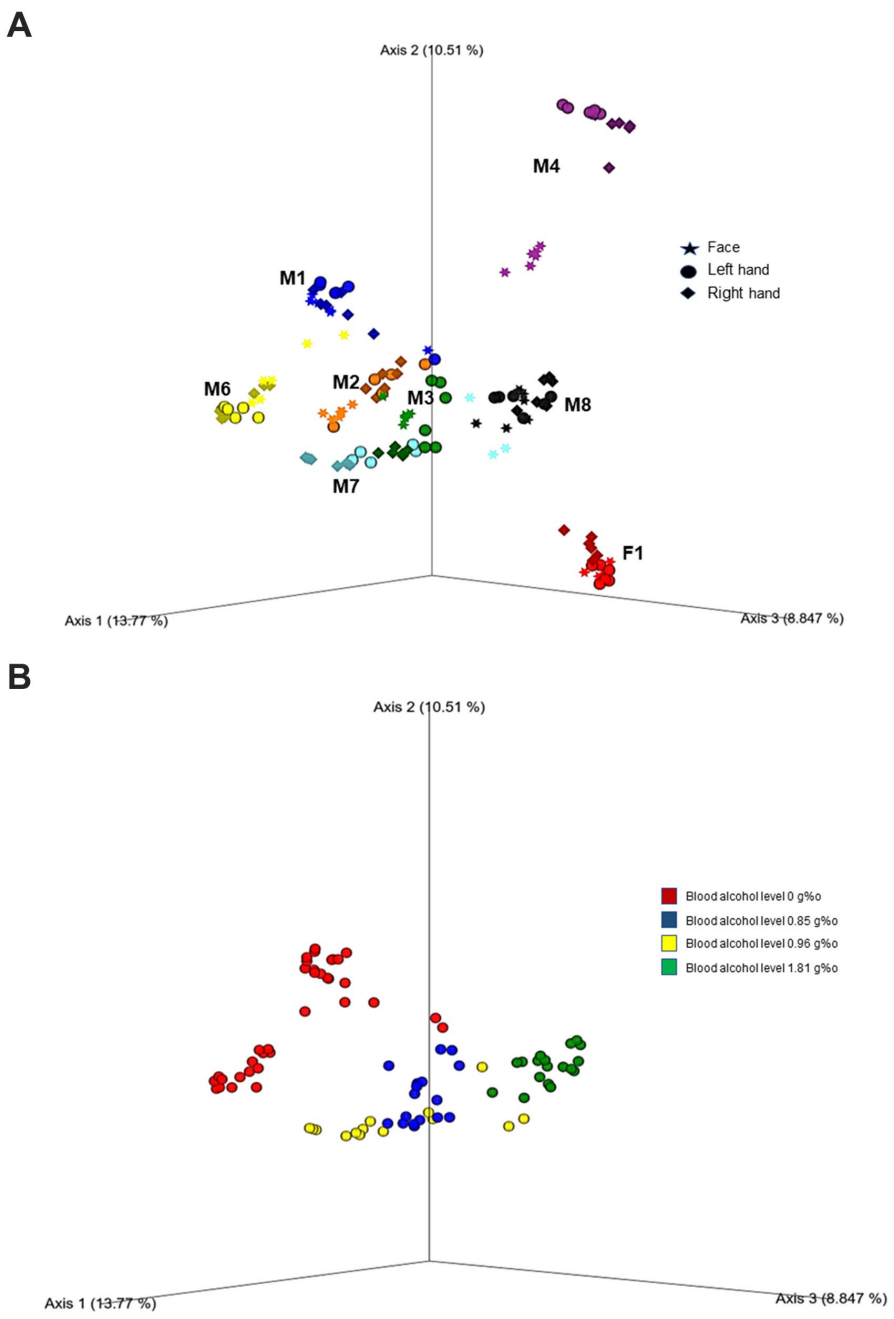
Principal coordinate analysis (PcOA) of the beta diversity (Bray Curtis) indicated significant clustering between cases **(A)** as well as toxicity (blood alcohol level) **(B)**. Further downstream Pairwise PERMANOVA revealed different microbial diversity especially between the face and both hands in each case (*p* < 0.005).

Further, beta diversity analysis using the Bray-Curtis matrix revealed clear clustering and separation among cases, as demonstrated in Figure 2A (PERMANOVA, 999 permutations: Pseudo *F* = 19.89; *p* = 0.001). To assess the microbial diversity across different body areas (face, left hand, and right hand) for each case, we conducted pairwise PERMANOVA analysis. Interestingly, all cases showed significant separation, particularly between the face and both hands (*p* < 0.005). However, two cases (M2 and M8) showed no significant difference in beta diversity between the right and left hands (*p* > 0.05). In addition, we observed significant differences in microbial diversity based on both alcohol toxicology reports (Figure 2B; PERMANOVA, 999 permutations, Pseudo *F* = 12.41; *p* = 0.001) and the places where the bodies were discovered (PERMANOVA, 999 permutations, Pseudo *F* = 11.45; p = 0.001).

### Taxonomic diversity

A total number of 37 phyla, 116 classes, 347 orders, 691 families, and 1,761 genera, were taxonomically assigned for all cases and collection (body areas). Firmicutes was identified as the dominant phylum for M4 (>93.23% for all body sites), M8 (>78.39%) and F1 (>68.04%). The three cases had a similar cause of death which was myocardial fibrosis, while M4 and F1 shared a medical history of dementia. It is of note that M4 had multiple other medical conditions in addition to dementia, documented in the medical records (Supplementary Figure 1). Further, the face samples of M4 comprised a maximum abundance of 60.74% Actinobacteria, while in F1 this phylum accounted for less than 10%. For F1 there was not a notable difference in the microbiome of all sample types, while for M4 a slight difference could be observed between the sampling times T0 and T1.

M1 and M2 shared the medical history of being ethanol consumers, however M2 and M3 shared a more similar microbiome profile than M1 and M2. As such, M2 and M3 accounted for more Acidobacteria than other cases, with an average relative abundance of 24%, while M2 comprised the highest relative abundances averages of Fusobacteria (50%) and Verrucomicrobia (42%). According to the toxicological report, the highest blood alcohol level of 1.81 g% was recorded for M8, who showed a different profile than M1 and M2, with Firmicutes (98.40%) as the dominant phylum. Firmicutes (70.01%) and Actinobacteria (96.96%) prevailed in M6, followed by Proteobacteria (36.10%), with slight differences between T0 and T1 for all sample types.

When advancing to a lower taxonomic level (class), the differences in the microbiome patterns between cases become more visible. F1 is different than M4, even if they shared a similar medical background of dementia. F1 had Clostridia recorded in higher abundances (48.9%), as well as Gammaproteobacteria (13%), and Bacteroidia (7%), while extremely low abundances of Erysipelotrichia (1.78%; more on the hands than on the face samples), and Negativicutes (2.9%; more on the face than on the hands samples) were observed. M4 samples were not characterized by great diversity, while the hands samples microbiome exhibited a different profile than the face samples, being characterized by the predominance of Bacilli (96%).

M1, M2, and M3 shared a more similar microbiome pattern, with a lower relative abundance of Bacilli (24%) and a higher relative abundance of Actinobacteria (27%). M3 profile showed higher relative abundances of Negativicutes (12.7%), more abundant on the face samples than on the samples from the hands. M3 microbial pattern appeared to be more similar to M2. Alphaproteobacteria was present in all three cases, with an average abundance of 15%, while Fusobacteria was observed in M2 and M3 accounting for only 10%. M7 microbiome profile was similar to M6, with high percentages recorded for Actinobacteria (13%) and Gammaproteobacteria (4%).

At the order level, the microbial profiles began to be increasingly diverse and case-specific, more noticeable for M2, M3, and M1, followed by M6, M7, and M8, in this order, respectively. Bacillales representatives were present in all cases, with the lowest relative abundances recorded for F1 (1.96%) and the highest relative abundances registered for M4 (96.84%). Lactobacillales was observed in all cases as well, with exceptionally low abundances in M1 (>1%).

F1 profile was more distinctive than all M cases, having Clostridiales (37%) and Lactobacillales (33%) dominating in all sample types and collection times, and exhibiting a higher diversity for MS (left hand) and MD (right hand), for T1 collection time. F1 also recorded the highest percentages of Enterobacteriales (>10%) from all cases. Micrococcales exhibited the highest relative abundances in M1 (13%) and M6 (8%). Propionibacteriales was dominant for the face samples of M4 (21%), while Betaproteobateriales prevailed in M6 (10%) and M8 (13%) face samples, for T0 collection time. Corynobacteriales was more prevalent in the overall samples of M1 (24%) and M6 (17%).

The microbiome diversity was even more case-specific at the family rank, for M3, M2, and M1. f representatives were present in most samples, at a constant abundance of 10% in F1. Pseudomonadaceae was more prevalent in M1 (37.5%) and M2 (12%) samples, while Staphylococcaceae predominated in the left hand (MS) of M4 (93.35%), being present in all cases, with the lowest relative abundances recorded for F1 (<1%). Neisseriaceae was prevalent in the hand samples of M6 (13.33%), for T1 collection time. Corynebacteriaceae was more abundant in M1 (44.81%) and the face samples of M6 (62.44%).

Regarding the genera taxonomic consistency among the investigated samples (Supplementary Figure 2), *Staphylococcus* sp. was present in all samples, with the highest abundances recorded for M4 (96.81%) and M8 (95.99%). Following, *Streptococcus* sp. was present in most samples, with the highest abundances recorded for M7 (32.01%). *Clostridium sensu stricto* was the most predominant genus in the face samples of M7 (83.06%), for T0 collection time. *Lactobacillus* sp. prevailed in F1 (38.91%), together with *Escherichia—Shigella* (10%), *Ruminococcus* sp. (10%) and *Sarcina* sp. (11.75%). M1 showed *Brevibacterium* sp. (>10%) and *Pseudomonas* sp. (37.50%). *Gemella* sp. was recorded in higher abundances in M6 (18.39%) and M7 (15.73%), while *Cutibacterium* sp. was prevalent in M4 (38.75%). *Ignatzschineria* sp. was observed in abundances as high as 9.47% in the face samples of M1.

### Skin microbiome differences between collection times

**Figure 3:**
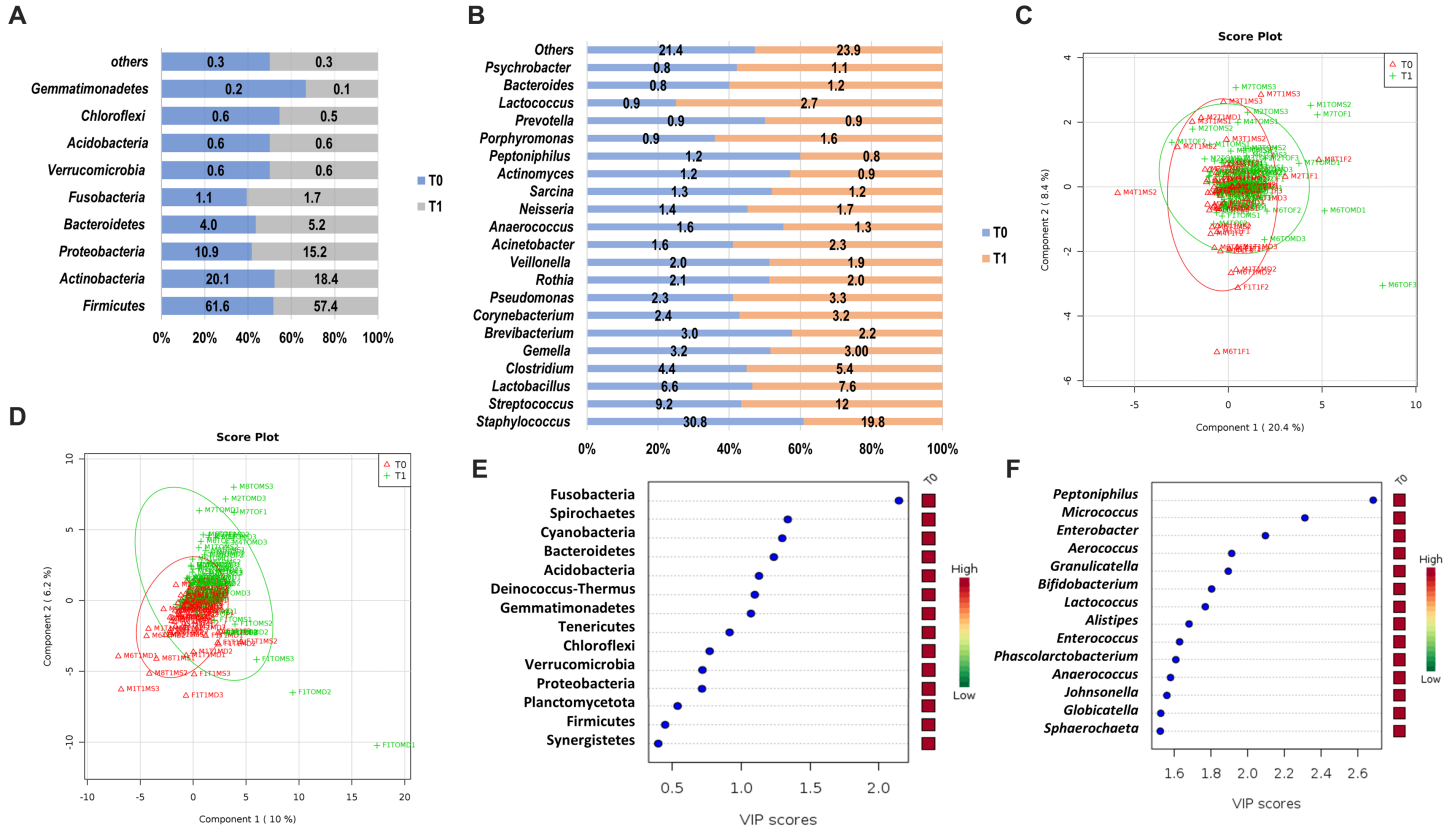
The structure of skin microbiome upon arrival at the morgue (T0) and after 12 h interval period (T1) at phylum and genus level. Relative abundance of bacteria phyla **(A)** and genera **(B)** at T0 and T1. Data are represented as mean relative abundance; only more than 0.01% is shown. **(C)** PLSDA score plot shows a similar community structure at the phylum level between T0 (red color) and T1 (green color). **(D)** A discriminant pattern between the microbiome of T0 and T1 was observed on the PLSDA score plot. The line ellipses on the PLSDA score plots indicate the 95% confidence interval. **(E)** Seven key phyla in T0 and T1 with VIP scores of more than 1.0 were identified. **(F)** Several key genera with the highest VIP scores were identified. The color key represents the relative abundance of the identified phyla/genera in relation to its important attributes in the discriminant pattern of skin microbiome on the PLSDA model.

We observed a similar composition of bacteria phyla on the skin microbiome of the cadaver upon arrival at the morgue (T0) and after 12 h (T1; Figure 3). The most abundant phyla observed in both sampling periods were Firmicutes (T0, 61.6%; T1, 57.4%), followed by Actinobacteria (T0, 20.1%; T1, 18.4%), Proteobacteria (T0, 10.9%; T1, 15.2%) and Bacteroidetes (T0, 4.0%; T1, 5.2%; Figure 3A). These prevalent phyla represent 87.6 and 96.2% of skin microbiota composition in T0 and T1, respectively. The other phyla had a relative abundance of less than 2.0%. Comparative analysis of the abundance of each phylum revealed that Firmicutes and Actinobacteria were higher in T0 than T1. In contrast, Proteobacteria and Bacteroidetes were higher in T1 than in T0 (Figure 3A). Two phyla/genera had relative abundance two folds higher in T1 than T0: Fusobacteria (2.24-fold) and *Deinococcus* (2.22-fold). However, there was no significant difference in the skin microbiota composition of the phyla as mentioned above, except for Fusobacteria, which was observed statistically significantly higher in T1 (1.7%) than upon arrival at the morgue (T0, 1.1%; *p* = 0.049).

At the genus level, *Staphylococcus* were prevalent and observed highest in T0 (30.8%) as compared to T1 (19.8%; Figure 3B). However, other prevalent genera such as *Streptococcus* (T1, 12.0% vs. T0, 90.2%)*, Lactobacillus* (T1, 7.6% vs. T0, 6.6%) and *Clostridium* (T1, 5.4% vs. T0, 4.4%) were observed higher in T1 as compared to T0. These prevalent genera represent 51.0% and 44.8% of skin microbiota composition at the genus level in T0 and T1, respectively. The other genera had a relative abundance of less than 3 %. Fold change analysis found that several genera had an abundance two folds higher in T1, such as *Jeotgalicoccus* (5.62-fold), *Pseudoclavibacter* (3.32-fold), *Granulicatella* (3.20-fold), *Malassezia* (3.10-fold), Solirubrobacter (2.87 fold), *Mobiluncus* (2.37 fold), *Deinococcus* (2.33 fold), *Cardiobacterium* (2.22 fold), *Peptoniphilus* (2.19 fold), *Sporosarcina* (2.18 fold), *Olsenella* (2.10 fold), and *Gemella* (2.07 fold). However, only three genera had statistically significant abundance differences between T0 and T1. Genus *Peptoniphilus* was significantly higher in T0 (*p* = 0.005), whereas *Micrococcus* and *Enterobacter* were significantly higher in T1 (*p* = 0.016; *p* = 0.029), respectively.

A PLSDA analysis observed weak and moderate discriminant patterns between skin microbiome at phyla level (R^2^ = 0.14) and genus level (R^2^ = 0.57) for T0 and T1, respectively (Figures 3C,D). From the VIP analysis, abundances of seven phyla with a VIP score of more than one was responsible for the discriminant pattern of skin microbiome between T0 and T1 (Figure 3E). At the genus level, 55 genera contributed to the clustering pattern observed on the skin microbiome between T0 and T1 with relative abundances of *Peptoniphilus*, *Micrococcus* and *Enterobacter,* the most important factor with VIP scores of more than 2.0 (Figure 3F).

### Skin microbiome differences between cases and cause of death

**Figure 4:**
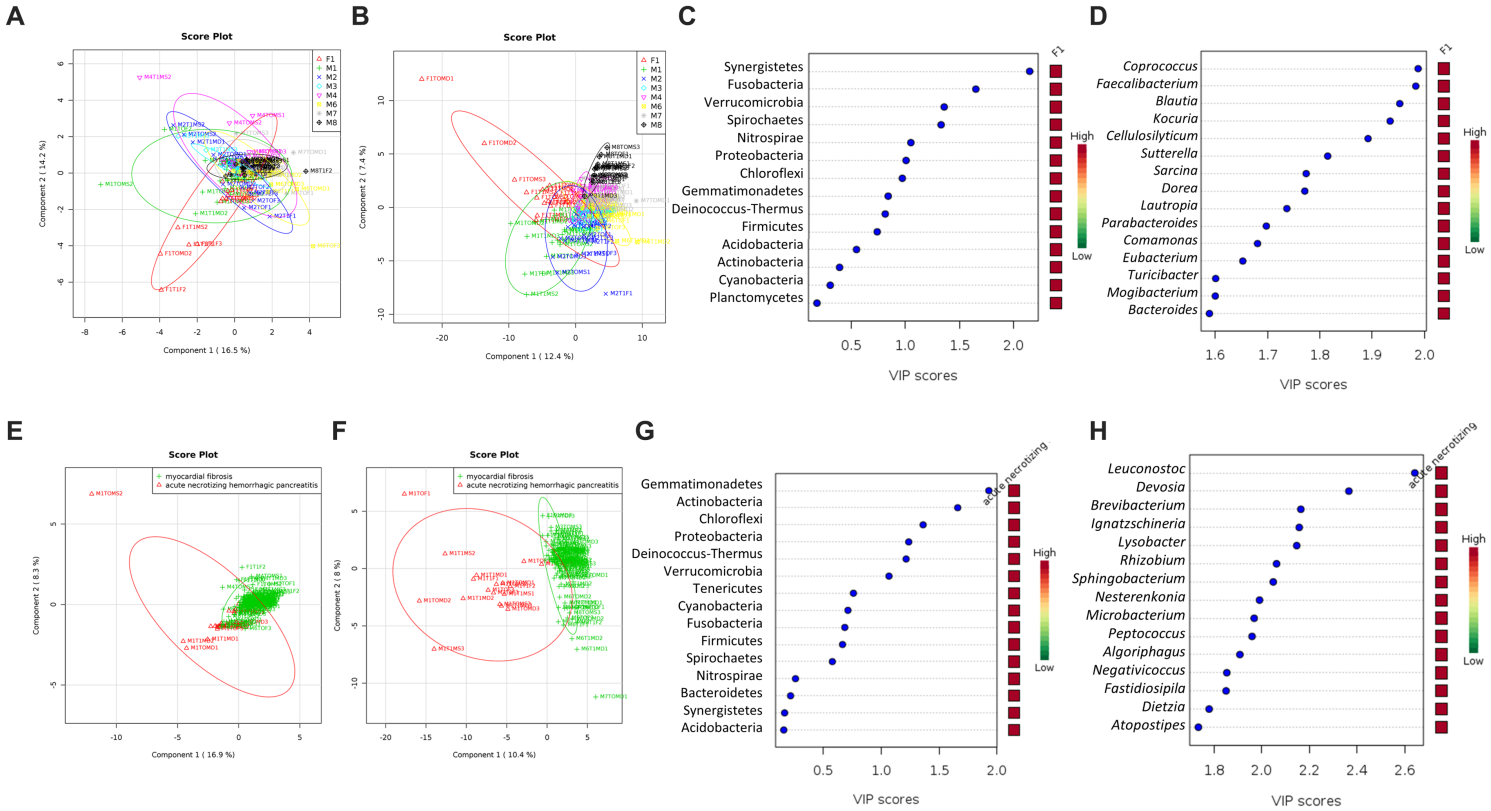
The community structure of the skin microbiome between cases and causes of death (acute necrotizing hemorrhagic pancreatitis vs. myocardial fibrosis). PLSDA score plot shows a distinct community structure between cases at the phylum level **(A)** and genus **(B)**. Data label (Case F1, red; M1, green; M2, blue; M3, cyan; M4, pink; M6, yellow; M7, gray; M8, black). The line ellipses on the PLSDA score plots indicate the 95% confidence interval. **(C)** Seven key phyla with a VIP score of over 1.0 were identified. **(D)** Several key genera with the highest VIP scores of more than 1.5 were identified. **(E)** PLSDA score plot shows a discriminant pattern at the phylum level **(E)** and genus **(F)** between acute necrotizing hemorrhagic pancreatitis (M1, red color) and myocardial fibrosis (F1, M3, M4, M6, M7, M8, green color). Data for M2 was excluded due to no information on the cause of death. Several key phyla **(G)** and genera **(H)** with VIP scores of more than 1.5 were identified. The color key represents the relative abundance of the identified phyla/genera in relation to its important attributes in the discriminant pattern of skin microbiome on the PLSDA model.

The skin microbiome profiles were distinct between cases at the phylum level (R^2^ = 0.43; Figure 4A) and were observed to be more substantial at the genus level (R^2^ = 0.84; Figure 4B). At the genus level, the skin microbiome profile of cases F1 and M1 were separated from the other cases (M2 to M8; Figure 4B). Based on the VIP score, Synergistetes, Fusobacteria, Verrucomicrobia, Spirochaetes, Nitrospirae, and Proteobacteria are the important phyla with a VIP score over than 1.0 (Figure 4C). However, 52 genera had a VIP score of more than 1.0, of which 17 genera had a VIP score of more than 1.5, thus these genera could be considered the most important genera (Figure 4D). These phyla and genera are the major variables contributing to the skin microbiome clustering pattern between cases, as observed on the PLSDA score plot (Figures 4A,B).

All six phyla with VIP scores over 1.0 (Figure 4C) were among the 12 phyla with statistically significant differences in relative abundances in at least one of the cases (Table 2). The phylum Synergistetes was observed to be highest in F1 compared to the other cases (Table 2). In comparison, Actinobacteria and Proteobacteria were observed highest in two cases, M1 and M6 (Table 2). M1 had an elevated abundance of Chloroflexi and Deinococcus-Thermus than the other cases (Table 2). For M2, Tenericutes were observed to increase significantly (Table 2). As previously observed, Tenericutes correspond to bloat stage (Adserias-Garriga et al., 2017). Further studies regarding the presence of these taxa are required to understand the postmortem abundance dynamics. In M6, the relative abundances for Fusobacteria were elevated significantly compared to the other cases (Table 2).

**Table 2:** Abundance of bacterial taxa that differed between cases.

Bacterial taxa	*P*-value	Tukey *Post hoc* tests comparison
*Synergistetes*	7.4048E − 14	F1 > M1; F1 > M2; F1 > M3; F1 > M4; F1 > M6; F1 > M7; F1 > M8
*Actinobacteria*	4.8491E − 09	M1 > F1; M6 > F1; M1 > M2; M1 > M3; M1 > M4; M1 > M7; M1 > M8; M6 > M2; M6 > M3; M6 > M4; M6 > M7; M6 > M8
*Fusobacteria*	2.29E − 06	M6 > F1; M6 > M1; M6 > M2; M6 > M3; M6 > M4; M6 > M8
*Firmicutes*	2.4487E − 06	M4 > M1; M4 > M2; M8 > M2; M4 > M3; M4 > M6; M4 > M7
*Proteobacteria*	4.2215E − 06	M1 > M2; M1 > M3; M1 > M4; M1 > M7; M1 > M8; M6 > M2; M6 > M3; M6 > M4; M6 > M7
*Tenericutes*	8.13E − 05	M2 > F1; M2 > M1; M2 > M3; M2 > M4; M2 > M6; M2 > M8
*Verrucomicrobia*	0.0017727	M4 > M6; M4 > M7; M4 > M8
*Bacteroidetes*	0.0022147	F1 > M4; M6 > M3; M6 > M4
*Spirochaetes*	0.0069456	M6 > M1; M6 > M4
*Chloroflexi*	0.02042	M1 > F1; M1 > M7; M1 > M8
*Deinococcus-Thermus*	0.04433	M1 > M2

>, higher in relative abundance (%), Significant *P* < 0.05 by ANOVA and Tukey *Post hoc* test. Refer to Table 1 for the metadata of each case.

At the genus level, 130 genera had significant differences in relative abundance in at least one of the cases (Figure 4D). All 17 genera with the highest VIP score over 1.5 were included. Out of the 130 genera, a total of 21 genera were elevated significantly in F1 compared to other cases. Two genera, *Collinsella* and *Sporosarcina,* were elevated significantly in M1 only.

A further comparison of the skin microbiome between causes of death revealed a distinct grouping between cases with acute necrotizing hemorrhagic pancreatitis and myocardial fibrosis at phylum (R^2^ = 0.47) and genus (R^2^ = 0.90) levels (Figures 4E,F). From the VIP score analysis, there are six phyla with scores more than 1.0 which are Gemmatimonadetes, Actinobacteria, Chloroflexi, Proteobacteria, Deinococcus-Thermus and Verrucomicrobia (Figure 4G). The t-test analysis revealed that the skin microbiome of the case with acute necrotizing hemorrhagic pancreatitis had higher abundances of Gemmatimonadetes (*p* = 1.98E-07), Actinobacteria (*p* = 1.03E-05), Chloroflexi (*p* = 3.57E-04), Proteobacteria (*p* = 0.001), Deinococcus-Thermus (*p* = 0.002), and Verrucomicrobia (*p* = 0.006) than the cases with myocardial fibrosis. These phyla were elevated more than two-fold in the case with acute necrotizing hemorrhagic pancreatitis. The highest was Chloroflexi (FC = 18.44), followed by Deinococcus-Thermus (FC = 9.48), Gemmatimonadetes (FC = 4.14), Actinobacteria (FC = 3.53), Proteobacteria (FC = 2.94) and Verrucomicrobia (FC = 2.94).

At the genus level, there were 40 genera with VIP scores higher than 1.0, and 25 of these genera had the highest scores, more than 1.5 (Figure 4H). There were 61 genera that had significantly higher relative abundance in the skin microbiome of acute necrotizing hemorrhagic pancreatitis case as compared to the myocardial fibrosis cases. The most important 25 genera identified from VIP analysis were also among the 61 genera that had significantly elevated in skin microbiome of cadaver with acute necrotizing hemorrhagic pancreatitis. Among these 25 genera, five genera had elevated more than 100-fold in the skin microbiome of acute necrotizing hemorrhagic pancreatitis based on the fold change analysis. The highest is genus *Ignatzschineria* (FC = 1341.2), followed by *Leucobacter* (FC = 234.33), *Nesterenkonia* (FC = 230.11), *Negativicoccus* (FC = 117.53), and *Lysobacter* (FC = 105.95; Figures 4F–H). There were another 43 genera that have been elevated between two-to 100-fold in the cases with acute necrotizing hemorrhagic pancreatitis as compared to myocardial fibrosis.

## Discussion

We present here the outcomes regarding the skin postmortem microbial investigation of human bodies 12 h after morgue receiving. Most of the studies to date focused on investigating decomposition and microbiome in terrestrial environments (Tuomisto et al., 2013; Hauther et al., 2015), and over more extended periods of time, while the current study focused on the first 12 h upon morgue receiving of the bodies. This approach was used, since this is the typical timeframe for keeping cadavers at the morgue (at that facility), hence, trying to bring the current experimental research as close as possible to real cases. Sometimes, the collection of skin or tissue samples at the crime scene is not possible, therefore, only the morgue samples could be accessible. In a hypothetical case, limited-to-no information from the crime scene would be available to a forensic microbiologist, such as temperature and relative humidity records, living conditions, e.g., A better communication/collaboration between death investigators, forensic pathologists, and forensic microbiologists would help overcome difficulties and fill in the information gaps. Moreover, access to medical records and autopsy reports could prove to be crucial in understanding the microbial distribution after death.

As previously stated (Ahn and Hayes, 2021), an individual’s microbiome is influenced by both macroenvironmental factors (toxicological, location of discovery, socioeconomic, etc.) and microenvironmental factors (alcohol, diet, etc.); hence, this explains the specific necrobiome clustering based on medical backgrounds and cause of death, in the current experiment. Understanding the medical background would help tremendously in the identification of potential bacterial species as PMI estimators. Since the microbiome fulfills multiple functions within the living organism, like different metabolic functions, immune system development, and nutrient synthesis, researching the cause and manner of death, together with the dynamics of the decomposition process, will provide a clearer understanding of the microbiome distribution patterns throughout decomposition.

The built environment (location of body discovery; Gilbert and Stephens, 2018) is included within the macroenvironmental factors that can influence the microbial structure of each individual. In the present experiment, a distinct microbiome structure was observed between decedents found at an apartment building and other locations, such as hospital, stairway, and a house. Recognizing that the built environment has an impact on the individual’s microbiome (Sharma et al., 2019), the place of death must be considered when investigating skin postmortem microbial signatures. Among the microenvironmental factors, alcohol played an important role in understanding the microbiome distribution in the current experiment. Previous studies on animal models (Kantorski et al., 2007) showed an increase in *Streptococcus mutans* colonization correlated with 20% of alcohol intake. For the case with the highest alcohol concentration, M8, *Streptococcus* spp. was present, but did not represent a dominant taxon. Nevertheless, the results revealed a decrease in bacterial richness and diversity associated to an elevated blood alcohol concentration, similar to previous findings (Signoretto et al., 2010; Thomas et al., 2014). Liver cirrhosis has been associated with a unique increase in Fusobacteria (Chen et al., 2011), while in our experiment, an increase in abundance was observed in M8, representing the case associated with the highest alcohol concentration, 1.81 g%. It is of note that previous studies investigated the microbiome diversity linked with macro and microenvironmental factors in living individuals. Similarities between previous and current studies could be explained by the microbiome reflection of antemortem conditions during early PMI. Nevertheless, antemortem conditions (medical records, living environment, dietary factors) could play a key role in shaping the postmortem microbiome communities over a more extended postmortem period (Carter, 2020).

Previous data (Lutz et al., 2020) showed that the microbial beta diversity patterns could be of help in predicting the cause or manner of death. This is portrayed in the current research, as different microbial signatures have been associated with a specific cause of death, such as cardiovascular disease. Moreover, studies on heart thanatomicrobiome (Bell et al., 2018) revealed differences in bacterial diversity between males and females. Proteobacteria has been identified as predominant phylum for cardiovascular disease associated deaths (Metcalf et al., 2013; Shin et al., 2015). Kaszubinski et al. (2020) showed that death related to cardiovascular disease or natural causes present a higher beta diversity dispersion and a lower alfa diversity (Pechal et al., 2018; Pearson et al., 2019). However, age, other causes of death, lifestyle, must be considered when investigating beta and alfa diversities.

In one of these previous studies (Pechal et al., 2018) the living environment explained most of the microbial diversity. Actinobacteria and Bacteroidetes were observed to decrease during decomposition, while Proteobacteria presented an increasing trend. In our investigation, Actinobacteria and Firmicutes slightly decreased over the first 12 h, while Bacteroidetes and Proteobacteria increased. In previous studies Firmicutes was found to be a constant marker between different body areas (Roy et al., 2021), while this phylum and Proteobacteria were found to be the predominant taxa identified from buccal swabs collected from cadavers in different decomposition stages (Sguazzi et al., 2022; Ogbanga et al., 2023). The increase in Bacteroidetes is in contrast with previous findings (Pechal et al., 2018), however, the sampling areas must be taken into consideration (face and hands vs. eyes, nose, mouth). *Staphylococcus* and *Streptococcus*, common genera identified in living humans, prevailed in most of the analyzed skin samples, while being previously identified as dominant taxa associated with the mouth samples after more than 24 h after death (Pechal et al., 2018). As previously shown (Speruda et al., 2022) the presence of Bacilli, like *Staphylococcus* and *Streptococcus* species can also indicate an infection or a contamination. Furthermore, *Staphylococcus* and *Streptococcus* were identified (Hyde et al., 2013; Javan et al., 2016; Johnson et al., 2016; Adserias-Garriga et al., 2017; Pechal et al., 2018) as important PMI indicators by machine-learning algorithms, confirmed by our results, emphasizing the need for targeted studies on these two genera.

Prior studies (Sampson and Mazmanian, 2015; Sharon et al., 2016) revealed that the gut microbiome can play a role in the nervous system functions, thus, potentially influencing various nervous system disorders. In the current research, two cases were associated with medical records encompassing dementia, while one case out of the two presented additional medical conditions. Although the one case that had a clear medical record associated with dementia clustered separate of all samples, it is not clear if this is the sole factor, or if the sex could be another element to be considered. Nevertheless, in addition to the forensic implications, understanding the gut-brain microbiome-nervous system interactions could help in developing preventive therapies and treatments of various neurologic disorders. When Pechal et al. (2018) investigated the relationship postmortem microbiome—health status, a low microbial diversity was found to be associated with heart conditions, similar to our findings. A more complete set of medical conditions as reflected by the medical records could provide further insights into postmortem microbiome, as a chronic health condition or habit, like alcohol consumption, can impact the microbial biodiversity, as previously reported in living humans (Eckburg et al., 2005; Cabrera-Rubio et al., 2012).

Schmedes et al. (2017) identified *Propionibacterium acnes* as the species present in all body sites, while in the current study the *Propionibacterium* sp. was identified only in 86 samples, from the total of 150 analyzed. This difference can be explained by the sequencing approach, as Schmedes et al. (2017) used shotgun metagenomics. Moreover, the same authors observed that the hand samples yield accurate rates of classification, even though they contained fewer shared phylotypes between the two hands of the same individual. It is important to consider that the hands are the target of frequent recolonization by daily activities.

As Kodama et al. (2019) revealed that the postmortem skin microbiomes remain stable in the first 24 h, as in our study (in the first 12 h) with little to no variation. Moreover, these microbial communities show potential in establishing associations with personal objects, such as medical devices, mobile phones, keyboard, computer mice, and fabrics (Fierer et al., 2010; Meadow et al., 2014; Lax et al., 2015; Lee et al., 2016).

According to Oh et al. (2016), healthy individuals maintain their skin communities for up to 2 years (despite daily life perturbations). Pechal et al. (2018) suggested that the bacterial taxa in the first 24–48 h after death would represent antemortem bacterial communities.

It was previously discussed that two individuals are having in common only 13% of all identified phylotypes (Speruda et al., 2022), so focusing the research on shared taxa/patterns could lead to a better understanding of the role of microbes in decomposition. Among shared taxa, *Clostridium* spp. was observed during the first 72 h postmortem, up to the entire duration of decomposition (Plueckhahn, 1986; Javan et al., 2016, 2019), similar to our study, where *Clostridium sensu stricto* was predominant for the face samples. However, the representatives of this genus have been observed mostly in organ samples toward the end of the bloat stage (Hyde et al., 2013, 2015; Can et al., 2014; DeBruyn and Hauther, 2017).

Differences in the thanatomicrobiome communities were recorded in male vs. female cadavers (Javan et al., 2016); *Pseudomonas* species being representative for females, while *Rothia* was found to be prevalent in males. In the current research such comparison is not supported, given the 1:7 female to male ratio, however, differences could be observed. The human microbiome is not only dynamic or sex dependent (Turnbaugh et al., 2007; Sguazzi et al., 2022), but it is also concentrated in certain areas of the body, constituting specific niches, such as the intestines (Roy et al., 2021). Our alfa diversity results showed differences between body areas as well. Therefore, it is crucial to investigate these specific niches, such as the skin postmortem microbiome, to better understand what intrinsic and extrinsic factors, macro, and microenvironments, are creating these variations, to identify common putative bacteria as PMI estimators. Moreover, as previously emphasized (Kaszubinski et al., 2020) the microbiome specificity for certain body sites is stable in the first 48 h, reflecting the antemortem conditions. Age is another factor affecting the microbiome during, and after death. Aging is considered to have an impact on the intestinal microbiome (Xu et al., 2019; Brooks et al., 2023), the diversity decreasing with age (Leite et al., 2021), while, Proteobacteria being observed in higher abundances in older living subjects. During the current experiment, Firmicutes was more abundant in cases involving older adults, however, these observations are made postmortem. As our study revealed, the postmortem skin microbiome has potential in understanding the cause and manner of death, if corroborated with the medical records data. Future experiments including the corroboration between chronological age, the number of associated diseases, the number of medications used, and the microbiome diversity would be of great help to provide additional information. A standardized sampling area(s) would help in further comparisons of the postmortem skin microbiomes even between continents. Depending on the body’s state of decomposition, the selection of a precise sampling location would be helpful to be included in a standardized sampling protocol (Metcalf, 2019). A standardized collection protocol calls the need of standardized protocols for DNA extraction, amplification, sequencing, and data analysis. Additionally, the time elapsed between death and the collection of samples must be considered. This is important since microbiome changes are dynamic, the diversity being influenced by the external environment, and the internal microenvironment. Due to the limited number of analyzed cases, the current research must be continued and expanded, as studies from different continents/countries are paramount for understanding the role and application of the microbiome in forensic investigations worldwide.

Samples representing face and hands skin microbiome were investigated from eight cases within 12 h upon arrival at the morgue. The taxonomic characteristics of the skin microbiota varied across different body sites, with no significant differences in taxonomic profiles between the two collection times (T0 and T1). The investigation also showed that different microbial signatures could be associated with a specific cause of death, such as cardiovascular disease, while an elevated blood alcohol level could be associated with a decrease in bacterial richness and diversity. Moreover, the places where the bodies were discovered seemed to play an important role in explaining microbiome diversity.

Given the predictive patterns and ubiquitousness of microbes, these taxa have great potential to be used as PMI estimators. Human skin is personalized, however, some microbes were shared between cases, like *Staphylococcus* and *Streptococcus*. As many bodies are discovered in the first 48 h after death, knowledge of skin microbial diversity would constitute a powerful tool for the PMI estimation, in cases where additional methods cannot be used. The resulted outcomes provided an overview on the postmortem skin microbiome in the 12 h upon morgue receiving of the bodies; extensive future studies are required to complement the current data, including the investigation of a greater number of cases.

## Data availability statement

The original contributions presented in this study are publicly available. This data can be found here: https://www.ncbi.nlm.nih.gov/, PRJNA940997.

## Ethics statement

The studies involving human participants were reviewed and approved by Institute of Legal Medicine Iași (no. 26067/03.23.2021) and the Institutional Biosafety Committee (IBC) University of North Dakota (UND; no. IBC-202111-011). Written informed consent for participation was not required for this study in accordance with the national legislation and the institutional requirements.

## Author contributions

LI performed the experimental design, data interpretation, and wrote the manuscript. VJ performed sample and metadata collection. AM, SA, and LI analyzed and interpreted the raw data. All authors contributed to the article and approved the submitted version.

## Funding

This research received financial support from the Start-up research funding (LI), College of Arts and Sciences, University of North Dakota.

## Conflict of interest

The authors declare that the research was conducted in the absence of any commercial or financial relationships that could be construed as a potential conflict of interest.

## Publisher’s note

All claims expressed in this article are solely those of the authors and do not necessarily represent those of their affiliated organizations, or those of the publisher, the editors and the reviewers. Any product that may be evaluated in this article, or claim that may be made by its manufacturer, is not guaranteed or endorsed by the publisher.
