# Supplementary material for: Postmortem skin microbiome signatures associated with human cadavers within the first 12 h at the morgue
Source: Front Microbiol. 2023 Jul 26;14:1234254. doi: 10.3389/fmicb.2023.1234254 (PMC10410280; doi:10.3389/fmicb.2023.1234254)
Supplement: Supplementary file 1 [file Image_1.pdf]

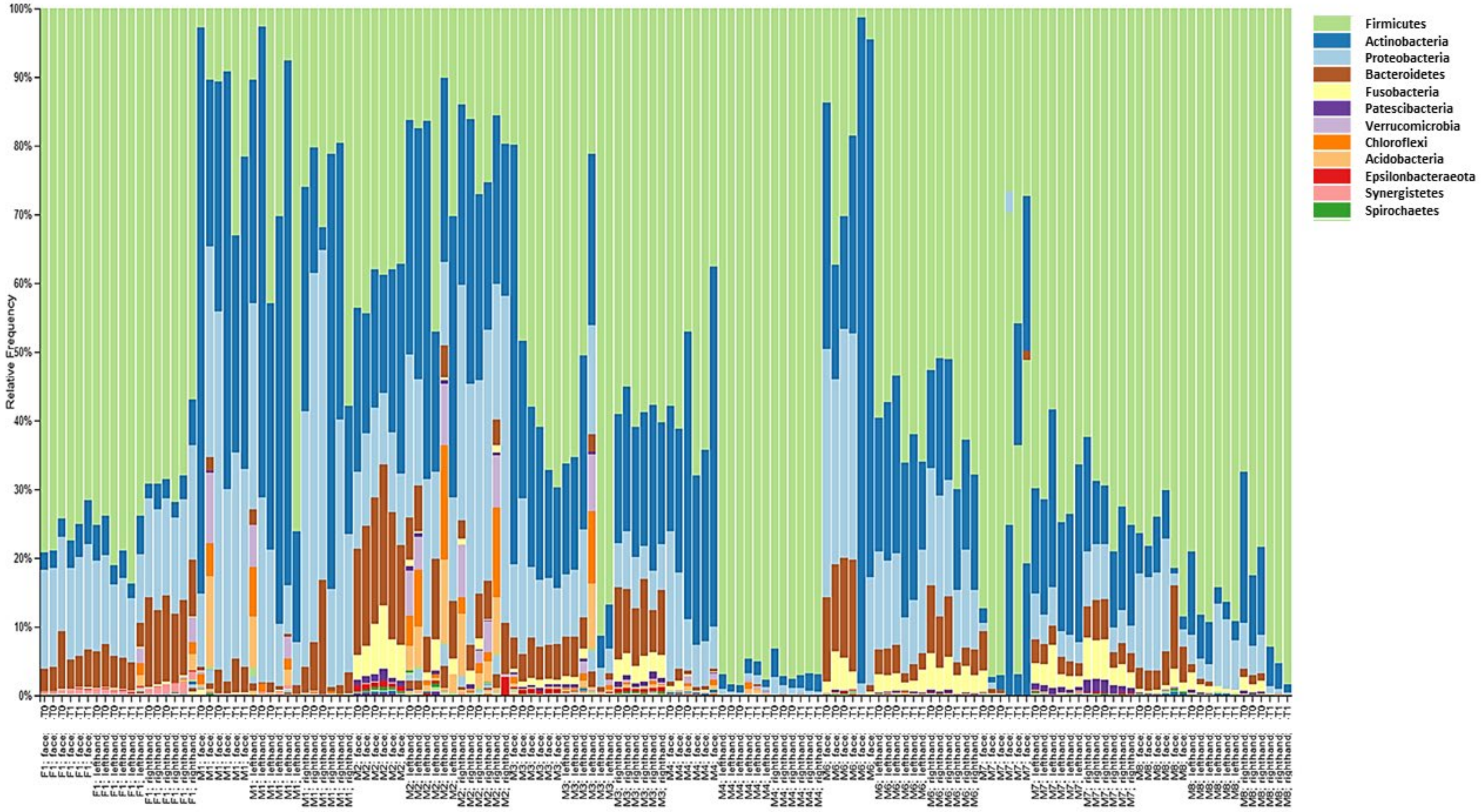

Supplementary Figure 1. Bacterial community composition (Phyla) for the postmortem skin-associated samples.
