# Supplementary material for: Postmortem skin microbiome signatures associated with human cadavers within the first 12 h at the morgue
Source: Front Microbiol. 2023 Jul 26;14:1234254. doi: 10.3389/fmicb.2023.1234254 (PMC10410280; doi:10.3389/fmicb.2023.1234254)
Supplement: Supplementary file 2 [file Image_2.pdf]

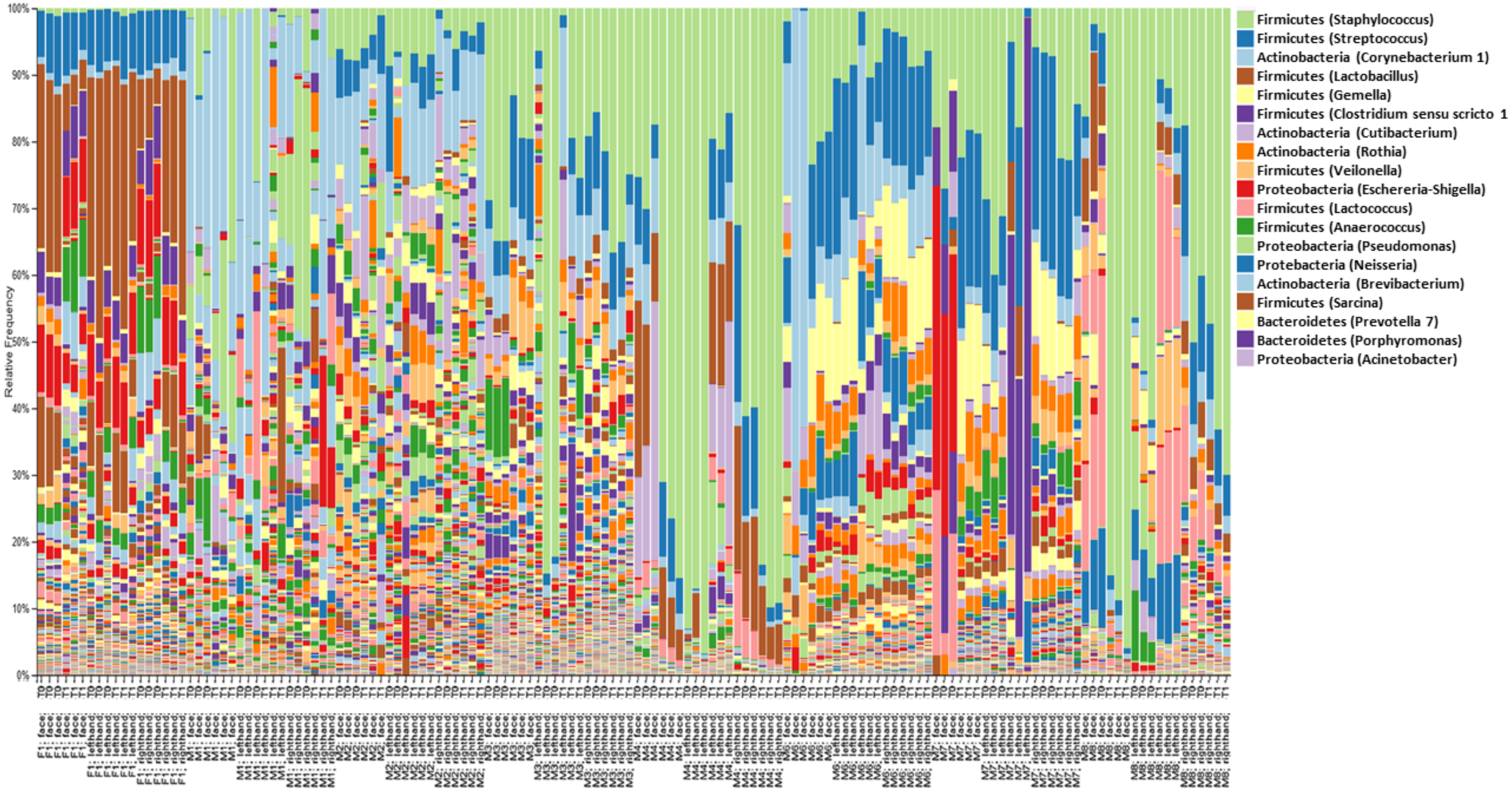

Supplementary Figure 2. Bacterial community composition (Genera) for the postmortem skin-associated samples.
